# Quality assurance of radiotherapy in the ongoing EORTC 22042–26042 trial for atypical and malignant meningioma: results from the dummy runs and prospective individual case Reviews

**DOI:** 10.1186/1748-717X-8-23

**Published:** 2013-01-30

**Authors:** Mehtap Coskun, William Straube, Coen W Hurkmans, Christos Melidis, Patricia F de Haan, Salvador Villà, Sandra Collette, Damien C Weber

**Affiliations:** 1 From the Departments of Radiation Oncology, Ankara Oncology Hospital, Ankara, Turkey; 2 From the Departments of Radiation Oncology, Washington University in St. Louis, St. Louis, USA; 3 From the Departments of Radiation Oncology, Catharina Hospital, Eindhoven, The Netherlands; 4 QA Strategic Committee, EORTC, Brussels, Belgium; 5 From the Departments of Head Quarter, EORTC, Brussels, Belgium; 6 From the Departments of Radiation Oncology, VU University Medical Center, Amsterdam, The Netherlands; 7 From the Departments of Institut Català d’Oncologia, HU Germans Trias, Badalona, Catalonia, Spain; 8 From the Department of Radiation Oncology, Geneva University Hospital, Radiation Oncology, Geneva, CH-1211, Switzerland

**Keywords:** Radiotherapy, EORTC, Quality assurance, Meningioma, Dummy run, Individual case review

## Abstract

**Background:**

The ongoing EORTC 22042–26042 trial evaluates the efficacy of high-dose radiotherapy (RT) in atypical/malignant meningioma. The results of the Dummy Run (DR) and prospective Individual Case Review (ICR) were analyzed in this Quality Assurance (QA) study.

**Material/methods:**

Institutions were requested to submit a protocol compliant treatment plan for the DR and ICR, respectively. DR-plans (*n*=12) and ICR-plans (*n*=50) were uploaded to the Image-Guided Therapy QA Center of Advanced Technology Consortium server (http://atc.wustl.edu/) and were assessed prospectively.

**Results:**

Major deviations were observed in 25% (*n*=3) of DR-plans while no minor deviations were observed. Major and minor deviations were observed in 22% (*n*=11) and 10% (*n*=5) of the ICR-plans, respectively. Eighteen% of ICRs could not be analyzed prospectively, as a result of corrupted or late data submission. CTV to PTV margins were respected in all cases. Deviations were negatively associated with the number of submitted cases per institution (p=0.0013), with a cutoff of 5 patients *per* institutions. No association (p=0.12) was observed between DR and ICR results, suggesting that DR’s results did not predict for an improved QA process in accrued brain tumor patients.

**Conclusions:**

A substantial number of protocol deviations were observed in this prospective QA study. The number of cases accrued per institution was a significant determinant for protocol deviation. These data suggest that successful DR is not a guarantee for protocol compliance for accrued patients. Prospective ICRs should be performed to prevent protocol deviations.

## Introduction

The objective of the European Organization for the Research and Treatment of Cancer (EORTC) 22042–26042 (NCT00626730) open study is to assess the impact of high-dose radiotherapy (RT) on progression-free survival, treatment tolerance and post-treatment global cognitive functioning in patients with a diagnosis of either atypical (World Health Organization [WHO] grade II) or malignant (WHO grade III) meningioma. The study flows and the details of the protocol have been described elsewhere [1]. The goal of Quality Assurance (QA) in RT is to reduce variabilities and uncertainties related to the different steps of treatment planning and actual patient irradiation, including but not limited to patient positioning and precise dose delivery to the target volume that may have an impact on tumor control or on the normal tissue toxicity [2]. As such, QA is of paramount importance when delivering high dose radiation to the brain in the context of a clinical trial. We report the results of the QA analysis of the first fully digital and prospective Individual Case Review (ICR) conducted in an international multicenter clinical trial for brain tumor.

## Materials and methods

### Radiotherapy protocol, treatment planning and QA requirements

Institutions were asked to perform a 1–3 mm planning Computed Tomography (CT) scan. Post-operative Magnetic Resonance Imaging (MRI) was compulsory. Target delineation was based on pre- and post-operative diagnostic imaging. Gross Tumor Volume (GTV) was defined as the visible tumor on fused images of Gadolinium-enhanced T1-MRI and planning CT-scan, including thickened dural trails and hyperostotic bone regions. The Clinical Target Volume (CTV) 1 (Simpson grade 1–3) and CTV2 (Simpson grade 4–5) had to include the GTV, with a margin of maximum 10 and 5 mm respectively, in order to account for microscopic disease extensions based on the preoperative tumor bed and peritumoral edema on imaging and pathology report. For patients with Simpson’s stage 1–3, who did not have residual tumor in the postoperative imaging, CTV was defined according to preoperative imaging and pathology report. The Planned Treatment Volume (PTV) -1 and PTV2 were defined as CTV1 and CTV2 plus a margin between 1–5 mm depending on the institutional protocols and treatment technique.

Three-Dimensional Conformal RT (3D-CRT) or Intensity-Modulated RT (IMRT) was delivered, according to discretion of the principal investigator. Nominal photon energies between 4 and 20 MV were used in fractions of 2 Gy, once per day. Independent from the WHO grading (i.e. II & III), 60 Gy to the PTV1 was delivered for all patients and for those with Simpson’s Stage >3, this treatment was followed with 10 Gy boost for PTV2 [1] The prescription point and dose homogeneity for each PTV was in accordance with ICRU 50/62 reports [3,4]. Lower and upper limits of cumulative doses for PTV were defined according to the dose received by the 95% (D95%) and 2% (D2%) of the PTV. To minimize under-dosage to the PTV, it was required that no less than 95% of the PTV should receive less than 95% of the prescribed dose.

Organs at risk (OAR) were delineated according to ICRU 62: brainstem, pituitary, cochlea, optic chiasm and optic nerves [4]. Recommended upper limit for the near maximum doses (D2%) were: 56 Gy for pituitary, 50 Gy for cochlea, 60 Gy for optic pathway structures, 64 Gy and 54 Gy for the brainstem surface and center, respectively.

### The digital central QA platform

The Dummy Run (DR)- and ICR-plans for all patients in this study were submitted to the Image-Guided Therapy QA Center of Advanced Technology Consortium (ITC-ATC) (http://atc.wustl.edu/). The following steps were followed for each DR and ICR: submission of the digital treatment planning data in appropriate format, fulfilling submission information form and sending an e-mail to ITC-ATC to indicate the submission.

### The DR and ICR

During the DR procedure, participating centers were requested to submit a protocol compliant treatment plan prior to trial activation. The EORTC QA level of complexity of this study was 4 [2]. As such, ICR was performed prospectively for each patient who had been accrued in the trial. The treatment planning data of all patients had to be reviewed per protocol within 5 days before the start of RT. The following protocol compliant digital data of all patients had to be submitted: planning images; structure contours; RT plan file; absolute 3D dose distribution (for each phase and sum of the treatment); color isodose images and dose-volume histograms for the total dose plan in absolute dose for target volumes and OAR.

### Plan evaluation

Two reviewers evaluated the plans (MC, DCW). Deviation parameters for tumor control and normal tissue toxicity are detailed in Table 1. For each target and OAR, a qualitative evaluation was made by inspection of the absorbed-dose distributions slice-by-slice to make sure that the PTV was adequately irradiated and OAR were adequately spared for each patient. Quantitative evaluation parameters for PTV indices are detailed in Table 2. Deviation parameters for PTV indices had not been used as protocol deviation parameter. The ICR-plan assessment was analyzed with the same DR criteria. Feedback was provided to the investigators to either confirm that the plan was protocol compliant or to recommend modifications if the plan was noncompliant. Revised plans were further assessed and, if necessary, additional changes were recommended. The QA deviations were also assessed as to whether they may have an adverse impact on tumor control or normal tissue toxicity.

**Table 1:** Parameters for plan evaluation

**PARAMETER**	**Recommended**	**Minor Deviation†**	**Major Deviation†**
***Parameters for tumor control***			
Dose Prescription	WHO grade II Simpson 1–3: 60 Gy	-	>60 Gy or <60 Gy
	WHO grade III or Simpson 4–5: 70 Gy	-	<70 Gy or >70 Gy
Dose Conformity	PTV D_95_% ≥ 95% of the prescribed dose	PTV D_95_% = 90 – 94% of the prescribed dose	PTV D_95_% < 90% of the prescribed dose
Target Volume Delineation	PTV covers all CTV and GTV	-	GTV and CTV outside the PTV*
	CTV covers all GTV	-	GTV outside the CTV
GTV to CTV margin	10 mm	11-15 mm	>15 mm
***Parameters for normal tissue toxicity***			
PTV D_2_% (Gy)	<107% of the prescribed dose	107-110% of the prescribed dose	>110% of the prescribed dose
Brainstem D_2_% or D_0.5cc_ (surface)	<64 Gy	64-65 Gy	>65 Gy
Brainstem D_2_% or D_0.5cc_ (center)	<54 Gy	54–55 Gy	>55 Gy
Optic Chiasm/Nerve D_2_%	<60 Gy	60-61 Gy	>61 Gy
Cochlea D_2_%	<50 Gy	50-51 Gy	>51 Gy

* *PTV for dose evaluation may be restricted to 5 mm below the patient outer contour.* † *Cases with both major and minor deviations were considered as with major deviation in overall analyses.*

***Abbreviations:*** PTV, *Planning Treatment Volume;* CTV*, Clinical Target Volume;* GTV*, Gross Tumor Volume;* D_95_%, D_2_%, *Dose received by 95 and 2 percent of the volume, respectively;* D_0.5cc_, *Dose received by 0.5 cc of the volume.*

**Table 2:** Quantitative parameters measuring quality: definition of indices

**Parameter [ref]**	**Definition**	**Optimal Value**	**Acceptable**	**Minor Violation**	**Major Violation**
RTOG Conformity Index (CI) [11]	*V*_*pi*_*/ V*_*T*_	1.0	1.0 – 2.0	≥ 0.9-1.0 or > 2.0-2.5	< 0.9 or >2.5
Target Coverage (TC) [12]	*V*_*T,pi*_*/ V*_*T*_	1.0	≥0.95 – 1.0	0.90 - 0.95	< 0.90
Homogeneity Index (HI)* [13]	*(D*_*2*_*%- D*_*98*_*%) / D*_*50*_*%*	0.0	0.0 - 0.12	0.12 - 0.2	> 0.2

*HI minor and major deviations defined according to protocol deviation parameters for dose conformity and PTV D_*2*_*%.*

***Abbreviations:*** V_pi_*, Prescription Isodose Volume;* V_T_, *Volume Target;* V_T,pi_*, 95% isodose volume within the target;* D_2_%, D_50_%, D_98_%, *Dose received by* 2, 50 *and* 98 *percent of the PTV, respectively.*

### Statistical method and considerations for correlation of planning evaluation parameters

Spearman correlation (*r*) and Fischer exact tests were used to determine the strength of the relationship between continuous variables and to determine the strength of the relationship between categorical variables, respectively. We used the Kappa coefficient to compare the agreement of the two reviewers. Statistical analyses were performed with SAS 9.3 (SAS Institute, Inc., Cary, NC), p < 0.05 was considered to be significant.

## Results

Twelve EORTC institutions from 7 European countries are currently participating in the EORTC 22042 study and have jointly included 54 patients until May 20^th^, 2012. Seven percent (*n*=4) of the cases could not be evaluated in QART review due to: non-submitted (*n*=2) or corrupted (*n*=2) data. Eighteen percent (*n*=9) of ICRs were performed retrospectively due to corrupted or late data submission. Five and 7 institutions were high- and low-recruiters (≥ 5 *vs.* < 5 patients), submitting 74% (*n*=37) and 26% (*n*=13) of the all accrued patients.

### Dummy run

The analysis of 12 DR-plans revealed 3 (25%) institutions submitting plans with major deviations that did not respect the requested RT dose of the trial: total dose for PTV1 was limited to 54 Gy and/or the required dose conformity for PTV1 was not protocol-compliant. Contrast was omitted in 41% (*n*=5) of the DR-plans from 5 institutions without any mentioned medical contraindication. Eighty-three percent (*n*=10) of the centers fulfilled the minimal dose constraints for the PTV (D95 > 95%). Target volume delineation parameters and maximum doses for the PTV and OAR were respected. Mean values for the PTV1 RTOG Conformity index (CI), Target Coverage (TC) and Homogeneity index (HI) (Table 2) were 1.4 (0.5 - 2.4), 0.8 (0.3 – 1.0) and 0.1 (0.1 - 0.3), respectively.

### Individual case review

All plans from 12 institutions delivered RT for a total dose of 60 Gy for PTV1 (*n*=50) and 70 Gy for PTV2 (*n*=7). Contrast was omitted in 24% (*n*=12) of the ICR-plans from 7 institutions without any mentioned medical contraindication. Fifty-six percent (*n*=28) of all treatments was planned with IMRT, while 44% (*n*=22) were planned with 3D-CRT. More than 4 fields were used in a majority (77%; *n*=17) of the 3D-CRT plans. Overall protocol deviations were observed similarly whatever the treatment technique used (3D-CRT, 32% deviation rate; IMRT, 32% deviation rate).

CTV/PTV dosimetry is summarized in Table 3. Mean PTV CI, TC and HI (Table 2) were 1.5 (0.6 - 2.8), 1.0 (0.5 – 1.0) and 0.1 (0.0 - 0.4), respectively. At least one major violation was observed in 18% (*n*=9) of the cases according to parameters in Table 2. PTV indices were found strongly inter-correlated (*r*=−0.41, p=0.0045 for CI-HI; *r*=−0.81, p<0.0001 for TC-HI and r=0.70, p<0.0001 for TC-CI). There was a negative correlation between PTV volume and CI value (*r*=−0.50, p=0.0005).

**Table 3:** Planning dose results for CTV and PTV in ICR-plans

**Target Volume**	**Mean Volume (cc), *±SD***	**Mean D_98_% (Gy), *±SD***	**Mean D_95_% (Gy), *±SD***	**Mean D_50_% (Gy), *±SD***	**Mean D_2_% (Gy), *±SD***
CTV1 (n=50)	105.5 ±83.2	58.3 ±2.2	58.9 ±1.5	60.6 ±0.8	62.5 ±1.0
CTV2 (n=7)	67.0 ±83.9	67.7 ±3	68.6 ±1.8	70.9 ±0.7	72.9 ±1.6
PTV1 (n=50)	172.6 ±106.4	55.7 ±4.6	57.0 ±3.7	60.4 ±0.8	62.7 ±1.1
PTV2 (n=7)	99.2 ±97.3	65.8 ±4.5	67.8 ±2.4	70.9 ±0.6	73.1 ±1.4

***Abbreviations:*** PTV, *Planning Treatment Volume;* CTV*, Clinical Target Volume;* D_98_%, D_95_%, D_50_%, D_2_%, *Dose received by 98, 95, 50 and 2 percent of the PTV, respectively.*

Mean volumes of brainstem, pituitary, cochlea, optic chiasm and optic nerve were 24.8±4.6, 0.4±0.2, 0.3±0.3, 0.9 ±0.5 and 0.7±0.3 cc, respectively. The relationship between CT slice thickness and OAR volumes showed positive correlation for optic chiasm (*r*=0.55, p<0.0001) and optic nerve (*r*=0.29, p=0.005) volumes. Mean values of the maximum dose (D2%, Gy) for brainstem, pituitary, cochlea and optic pathway were 16.0±2.0, 16.0 ±2.0, 10.0±15.0 and 18.0±2.0, respectively. Major deviations for normal tissue toxicity were observed in ICR-plans in which PTV consists of a part of the brainstem and/or cochlea or optic nerve.

**Figure 1:**
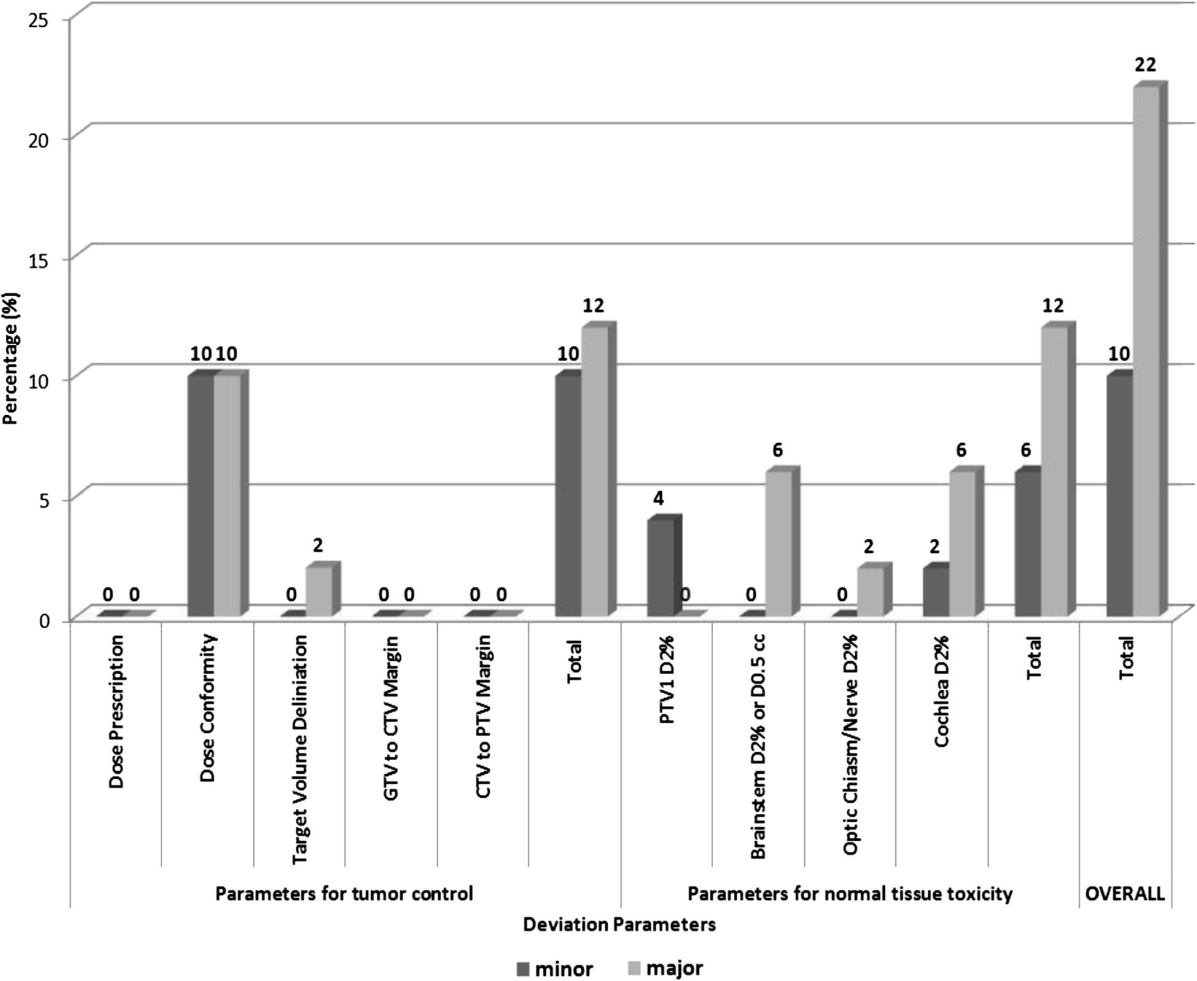
Observed deviations in EORTC 22042–26042 trial for Tumor Control and Normal Tissue Toxicity.

Deviations for tumor control and normal tissue toxicity in ICR-plans are illustrated in Figure 1. Major and minor deviations were observed in 22% (*n*=11) and 10% (*n*=5) in all ICR plans. The observed major deviations in this study had a potential impact (i.e. would have had a critical impact on patient’s outcome had the radiation plan been applied without corrections) on tumor control (TCP) and normal tissue complication probabilities (NTCP; Figure 1). Among major deviations with a TCP impact, all but one (2%) were related to dose conformity (Figure 1). Regarding NTCP, major deviations were observed on all OARs (Figure 1).

**Figure 2:**
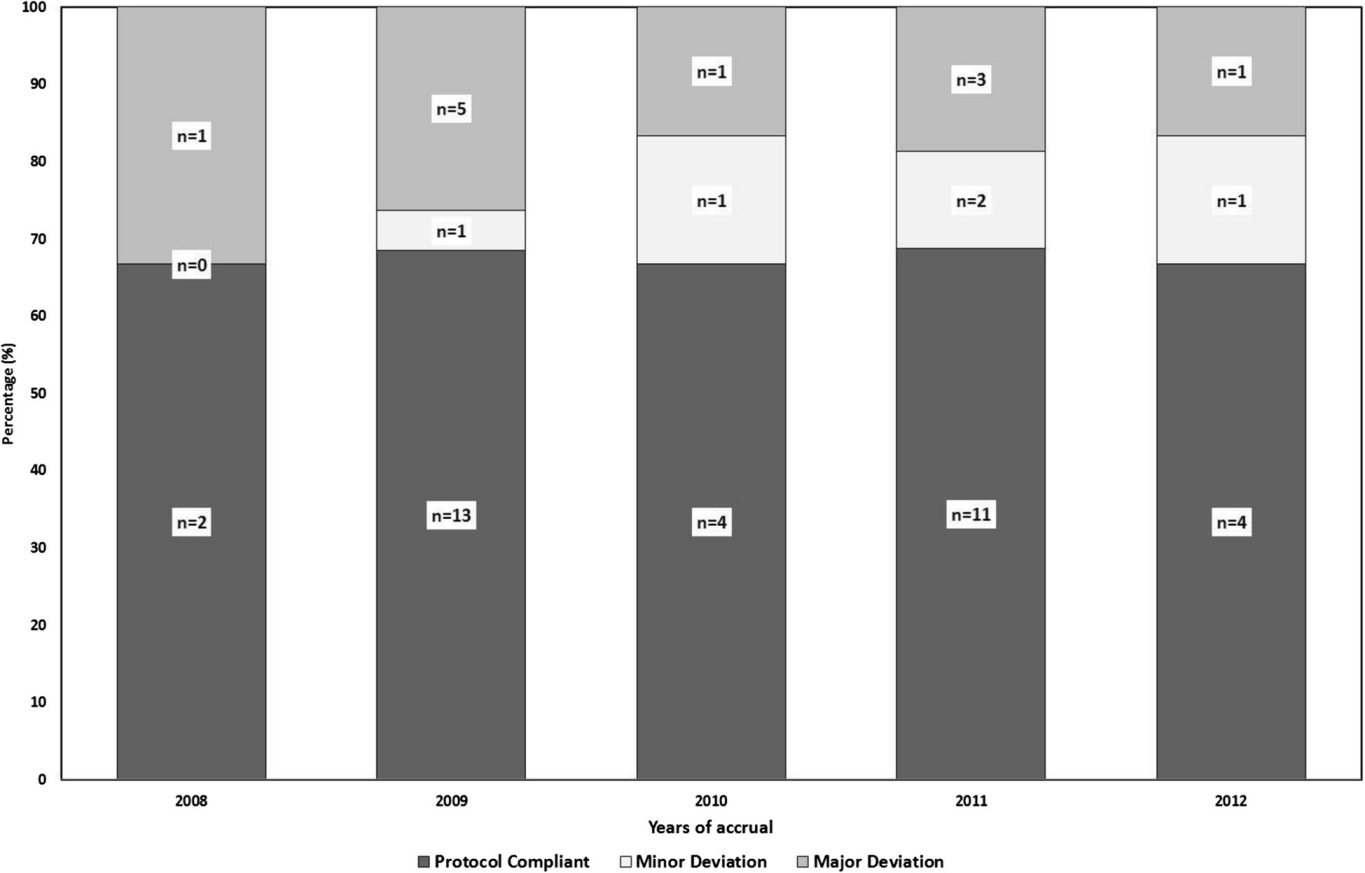
EORTC 22042–26042 Protocol-Compliance and -Deviations with Time.

A good inter-observer agreement was observed, as the two reviewers agreed in a majority (*n*=45; 90%) of the cases after discussion (K=0.50; 95%CI: 0.14 - 0.86; p=0.003). Fewer deviations were observed in plans stemming from high recruiting institutions (≥5 patients), when compared to those stemming from low recruiting centers (22% *vs.* 62%, respectively, p=0.007). Although major deviations were tend to decrease by the subsequent years of accrual, we did not observe an improvement in the protocol compliance with time, as a result of the increase rate of minor deviations (Figure 2).

No association between DR- and ICR-plan deviations was observed (p=0.12). Eighty-nine percent (*n*=8/9) of the institutions with no DR-deviation were found to have subsequently deviations with ICRs. Likewise, 67% (*n*=2/3) of institutions with DR-deviations did not have later any ICR’s deviations.

## Discussion

To the best of our knowledge, the present study is the first analysis of an interventional ICR QA procedure prospectively performed in a clinical RT brain trial (i.e. EORTC QA level 4 [2]). We have observed a substantial number of deviations in approximately one third of accrued patients that may have an impact on the primary-end point (Figure 1). The impact of protocol deviations on patient’s outcome [5], has been shown in a number of prospective studies [6-8]. Interestingly, the successful DR-plans did not guarantee protocol compliance during subsequent ICR submissions and that the overall deviation rate did not improve with time (Figure 2). As such, ICR should be prospectively performed in a clinical trial with RT.

Our report not only presents the data of this brain trial but also gives us opportunity to challenge the current QA paradigm. In previous studies, prospective reviews could not be conducted or were considered as ineffective [6,7,9]. We observed that only 7% of all ICRs could not be analyzed in this trial. A substantial number (18%) of cases were however retrospectively analyzed due to late submission or corrupted files during first submission to the digital QA platform. All (*n*=11) major deviations and 80% (*n*=4) of the minor deviations were detected by prospective review (*n*=41) and were discussed with institutions before the start of treatment. No significant association (p=0.19) was observed between the review type, prospective *vs.* retrospective, and the deviation detection rate, but these data should be cautiously interpreted due to the low number of patients in the retrospective review arm (*n*=9).

There was a significant association (p=0.007) between the number of accrued cases per institution and the number of observed deviations. Peters *et al.* reported similar findings in the prospective head and neck trial, with a cut-off of 5 patients *per* institution [8]. Duhmke *et al.* have also found similar results for early stage Hodgkin lymphoma, with a cut-off of 10 patients *per* institutions [10]. It would thus be appropriate to limit the inclusion of patients into prospective RT trials from reasonably high-accruing institutions (i.e. 5 – 10 patients *per* institution), so as to increase the protocol-compliance rate and possibly improve patient’s outcome [5].

The DR is designed to identify systematic planning or delivery RT errors and recognize protocol ambiguities before study treatment starts. This procedure assures that physicians understands the protocol requirement of a given trial, delineate target volumes and OARs appropriately, produce a protocol-compliant plan and are able to transfer the digital data to the QA platform [11]. Noteworthy, the DR deviation rate of 25% was observed at the beginning of the trial and an overall deviation rate of 32% was subsequently observed during patient’s accrual. As such, the DR procedure did not improve the protocol-compliance rate of the institutions, as no association between compliant- and non-compliant centers with deviation rates was observed. Parenthetically, the rate of i.v. contrast administration during RT planning for DR and ICR improved however from 56% to 76%. A DR-ICR correlation was observed in an EORTC prostate trial, but not in a low-grade glioma brain (EORTC 22033–26033) trial [11]. Moreover, protocol compliance did not improve within the trial accrual period (Figure 2). Clinical trials usually take several years to be completed, institutional physicists and physicians may change and have a high turn-over rate, especially so for low-recruiting institutions. This will consequently increase the probability of protocol guidelines misinterpretation and systemic planning errors. Due to the former issue, QA analysis by the intergroup QA team should always be performed by two reviewers and inter-rater agreement should be reported. In our study there was a significant association between the two reviewers (*K*=0.5). Discrepancy was observed for only 5 ICR-plans with minor deviations.

During our analysis, we computed CI and TC (Table 2) to take into account both non-target tissue and PTV [12,13]. HI was calculated for absorbed-dose distribution within the PTV according to ICRU 83 recommendations [14]. There was a negative correlation between the PTV volume and CI value for the ICR-plans (p=0.0005). Similar findings were previously reported by Musat *et al.* and Knöos *et al.*[15,16]. We did not observe however any association between CI value and tumor shape or OAR’s vicinity or between PTV volume and TC or HI (p=0.10). We recommend the prospective capture of the PTV indices in clinical trials, as plan evaluation parameters, for future survival/toxicity correlation analyses to define optimal and suboptimal values (minor/major violations) more accurately for the choice of ideal dosimetry.

In conclusion, we have observed a considerable number of protocol deviations that may have a substantial impact on tumor control or radiation-induced toxicity. In this trial, DR could not avoid protocol non-compliance subsequently for ICR submission. Prospective ICR should be conducted to prevent protocol deviations that may have an impact on tumor control and/or toxicity. A substantial number of ICRs could not be prospectively evaluated as *per* protocol.

## Abbreviations

EORTC: European Organization for the Research and Treatment of Cancer; RT: Radiotherapy; WHO: World Health Organization; QA: Quality Assurance; ICR: Individual case review; CT: Computed tomography; MRI: Magnetic resonance imaging; GTV: Gross tumor volume; CTV: Clinical target volume; PTV: Planned target volume; 3D-CRT: 3D-conformal radiotherapy; IMRT: Intensity-modulated radiotherapy; OAR: Organ at risk; ITC-ATC: Image-guided Therapy QA center of Advanced Technology Consortium.

## Competing interests

The authors declare that they have no competing interests.

## Authors’ contribution

DCW was responsible for the primary concept and the design of the study; MK and DCW performed the data capture and analysis. MK and DCW drafted the manuscript; SC performed the statistical analysis; MK and DCW reviewed patient data; all authors revised the manuscript. All authors have read and approved the final manuscript.
